# 

**Published:** 1837-07

**Authors:** 


### BRITISH AND FOREIGN

### MEDICAL REVIEW

OR

### QUARTERLY JOURNAL

OF

### PRACTICAL MEDICINE AND SURGERY

---

EDITED BY

JOHN FORBES M.D. F.R.S.

AND

JOHN CONOLLY M.D.

EDITORS OF THE CYCLOPÆDIA OF PRACTICAL MEDICINE

---

VOL. IV

S.G.O.L. INDEXED

JULY—OCTOBER 1837

Surgeon General's Office LIBRARY. 34527 Washington D.C.

LONDON

SHERWOOD GILBERT AND PIPER

PATERNOSTER ROW

MDCCCXXXVII

W
1
BR 54

LONDON:

PRINTED BY J. AND C. ADLARD, BARTHOLOMEW CLOSE.

these very few are medical. In regard to the duration of the courses, Dr. Guiette told me that his course of Physiology lasts the whole year; three lectures a week. The fees of the university are,—*Droit d'inscription*, 15 francs; *Retribution annuel*, 200 francs.

---

### BOOKS RECEIVED FOR REVIEW.

ENGLISH.

1. Homœopathy briefly explained, in a Letter to Sir Henry Halford, Bart. M.D. &c. By a Licentiate.—London, 1837. 8vo. pp. 66.

2. The Cyclopædia of Practical Surgery. Edited by W. B. Costello, M.D. Part I. April, 1837.—Royal 8vo. pp. 112. 5s.

3. Guy's Hospital Reports. No. IV. April, 1837. Edited by G. H. Barlow and J. P. Babington, A.M. 8vo. pp. 310; Plates. 6s.

4. A Companion to the Ship's Medicine Chest, &c. By W. G. Faddy, Surgeon.—London. 12mo. pp. 60. 2s. 6d.

5. A Manual of the Diseases of the Eye. By S. Littel, jun. M.D.—Philadelphia, 1837. 8vo. pp. 255.

6. A Practical Compendium of the Diseases of the Skin, including a particular Consideration of the more frequent and untractable Forms of these Affections; with Cases. By Jonathan Green, M.D. &c. Second Edition, corrected and improved; with two Plates.—London, 1837. 8vo. pp. 371. 12s.

7. A Manual of General Anatomy. By J. F. Meckel, &c. Translated from the German into French, by A. J. L. Jourdan and G. Breschet. Translated from the French, with Notes, by A. S. Doane, A.M. M.D., and others.—London, 1837. 8vo. pp. 421. 6s.

8. A Letter to Sir Henry Hardinge, on the Effects of Solitary Confinement on the Health of Soldiers in Warm Climates. By J. G. Malcolmson, Surgeon E. I. C. Service, late Secretary to the Madras Medical Board.—London, 1837. 8vo. pp. 23.

9. An Inaugural Discourse on Medical Eclectism. By J. C. Cross, M.D., Professor of Materia Medica in the Medical College, Ohio.—Cincinnati, 1835. Royal 8vo. pp. 20.

10. An Introductory Lecture, delivered in the Anatomical Theatre of the University of Maryland, on the 1st November, 1836. By R. E. Griffith, M.D. Professor of Materia Medica, &c.—Baltimore, 1837. 8vo. pp. 14.

11. A Letter from Dr. Brigham to D. M. Reese, M.D., author of Phrenology known by its Fruits.—Hartford (U. States), 1836. 8vo. pp. 24.

12. Observations on the Nature and Treatment of Calculous Diseases. By B. W. Dudley, M.D.—Lexington, 1836. 8vo. pp. 40.

13. A Treatise on the Diagnosis and Treatment of Diseases of the Chest. Part I. Diseases of the Lung and Windpipe. By W. Stokes, M.D. M.R.I.A., Physician to the Meath Hospital, &c.—Dublin, 1837. 8vo. pp. 557. 16s.

14. The Works of John Hunter, F.R.S., with Notes. Edited by J. F. Palmer, Surgeon. Vol. II., containing Treatise on the Teeth, with Notes by Thomas Bell, F.R.S.; and Treatise on the Venereal Disease, with Notes by G. G. Babington, Esq. — London, 1835. 8vo. pp. 448. 17s. 6d.

15. A Dictionary of Practical Medicine. By James Copland, M.D. F.R.S. &c. &c. Part IV.—London, 1837. 8vo. (From Fever to Heart.) Pp. 303. 9s.

16. Principles of the Theory and Practice of Medicine; including a third Edition of the Author's Work upon Diagnosis. By Marshall Hall, M.D. F.R.S. L. & E.—London, 1837. 8vo. pp. 503. 16s.

17. Medical Essays, by J. Hungerford Sealy, M.D. &c. No. II. The Imagination; its History and Effects.—London, 1837. 12mo. pp. 91. 3s.

18. An Address delivered to the Members of the Worcestershire Natural History Society, on the Opening of the Worcestershire Museum, Sept. 15, 1836. By Charles Hastings, M.D. F.G.S.—London, 1837. 8vo. pp. 57. 2s. 6d.

19. A Treatise on the Diseases and Injuries of the Larynx and Trachea; founded on the Essay to which was adjudged the Jacksonian Prize for 1835. By Frederic Ryland, Surgeon to the Town Infirmary, Birmingham.—London, 1837. 8vo. pp. 328; with six Plates. 18s.

20. The Teeth a Test of Age, considered with reference to the Factory Children. By E. Saunders.—London, 1837. 8vo. pp. 76.

21. An Exposition of the Symptoms, essential Nature, and Treatment of Neuropathy, or Nervousness. By J. M. Gully, M.D. &c.—London, 1837. 8vo. pp. 192. 6s.

22. Rudiments of Physiology. Part III. On Life, as manifested in Sensation and in Thought. By the late John Fletcher, M.D. Edited by R. Lewins, M.D.—Edinburgh, 1837. 8vo. pp. xxiii. 144. 4s.

23. The Cyclopædia of Anatomy and Physiology. Part X. Electricity—Eye. —R. 8vo. pp. 96. 5s.

24. A Treatise on Diet: with a View to establish, on practical Grounds, a System of Rules for the Prevention and Cure of the Diseases incident to a disordered State of the Digestive Functions. By J. A. Paris, M.D. F.R.S. Fifth Edition, corrected, enlarged, and nearly rewritten.—London, 1837. 8vo. pp. 414. 12s.

25. On the Nature and Treatment of Dropsical Diseases: in four Parts. Part I. and II. On Dropsies from suppressed Perspiration and diseased Kidney. Part III. On Dropsies from Impediment to the Circulation. Part IV. On Dropsies from Topical Affections. By J. Osborn, M.D., Fellow, and late President of the King and Queen's College of Physicians in Ireland. —Post 8vo. Second Edition. 7s.

26. An Exposition of the Signs and Symptoms of Pregnancy, the Period of Human Gestation, and the Signs of Delivery. Illustrated with numerous coloured Plates and Wood-cuts. By W. F. Montgomery, A.M. M.D. Professor of Midwifery to the King and Queen's College of Physicians in Ireland. In one volume.—8vo. 16s.

27. What Asylums are and ought to be: being the Substance of five Lectures delivered before the Managers of the Montrose Royal Lunatic Asylum. By W. A. F. Browne, Surgeon, &c.—Edinburgh, 1837. 8vo. pp. 231. 5s.

28. Additional Remarks on the Use of English Mineral Springs, especially those of Bath, Cheltenham, and Leamington; with the most recent Analyses. By E. Lee, Esq. M.R.C.S. &c.—London, 1837. 8vo. pp. 20.

29. Two Lectures on Lithotrity and the Bi-lateral Operation. By E. Lee, Esq. M.R.C.S.—London, 1837. 8vo. pp. 16.

30. The Transactions of the Provincial Medical and Surgical Association. Vol. V. —London, 1837. 8vo. pp. 527. 20s.

31. Transactions of the Philosophical and Literary Society of Leeds; consisting of Papers read before the Society. Vol. I. Part I.—London, 1837. 8vo. pp. 201; 12 Plates. 10s.

32. The Medico-Chirurgical Formulary, &c. By M. Ryan, M.D. &c.—London, 1837. 18mo. pp. 327. 3s. 6d.

33. Aphorisms on Natural and Difficult Parturitions, Puerperal Diseases, and on the Physical Management of Infants. By M. Ryan, M.D. &c.—London, 1837.—18mo. pp. 128. 2s.

FOREIGN.

1. Beretning, Betænkning og Indstilling fra en til at undersæge de sindsvages Kaar i Norge og gjære Forslag til deres forbedring in aaret 1825 naadigst nedsat Kongelig Commission. Udgivet af Fred. Holst, M.D., Prof. i Medecin, &c.—Christiania, 1828. 8vo. pp. 139.

2. Dissertatio de Acidi Nitrici usu Medico. Auctore Fred. Holst.—Christianiæ, 1816. 8vo. pp. 136.

3. Morbus quem Radesyge Vocant, quinam sit, qua namque ratione e Scandinavia tollendus? Commentatio. F. Holst, auctore.—Christianiæ, 1827. 8vo. pp. 157.

4. Universiteterne i Christiania og Upsala.—Christiania, 1836. 12mo. pp. 102.

5. Medicinal Taxt for Norge.—8vo. pp. 39. Christiania, 1834.

6. Fortegnelse over de auctoriserede Læger i Norge.—Christiania, 1834. 8vo. pp. 8.

7. Von den Wirkungen der gebräuchlichen metalle auf den menschlichen organismus überhaupt und als heilmittel und dem Kupfersalmiak-Liquor und andern Kupferpräparaten als solchen insbesondere. Von Dr. J. R. Köchlin.—Zurich, 1837. 8vo. pp. 186.

8. Die Mercurialkrankheit in allen ihren formen, geschichtlich, pathologisch, diagnostisch und therapeutisch dargestellt. Von G. L. Dietrich, M.D. &c.—Leipzig, 1837. 8vo. pp. 422.

9. Disquisitiones Sterilitatis muliebris. Auctore Joanne Person, M.D.—Petropoli, 1835. 8vo. pp. 90.

10. Handwörterbuch der gesammten Chirurgie und Augenheilkunde. Herausgegeben von Ernest Blasius, Professor der Chirurgie an der Universität zu Halle, &c. Erster Band. A—C.—Berlin, 1836.—8vo. pp. 884.

11. Das Heimweh und der Selbstmord. Von J. H. G. Schlegel, M.D. &c. 2 vols. —Hildburghausen, 1835. 8vo. pp. 125, 301.

12. Traité de Diagnostic et de Séméiologie. Par P. A. Piorry, M.D. &c.—Vol. I. et II.—Paris, 1837. 8vo. pp. 1300.

13. Bibliothek for Læger. No. IV., 1836; No. I. 1837. Redigeret af dens medlem C. Otto, M.D. &c.—Kjœbenhavn, 1836-7. 8vo.

14. De Fistula Urethro- et Vesico-vaginali Commentatio, remedia contra hunc morbum systematice et critice exponens. Auctore J. C. Bendz, M.D.—8vo. pp. 138. Hauniæ, 1836.

15. Memorie della Società Medico-Chirurgica di Bologna. Vol. I. Fasc. 1o. e 2o.—Bologna, 1835-6. R. 8vo. pp. 223.

16. Memorie sul Choléra-Morbus. Appendice al Bullettino delle Scienze Mediche di Bologna. Fasc. 1o. e 2o.—Bologna, 1836. 8vo. pp. 173.

